# ELMAS: a one-year dataset of hourly electrical load profiles from 424 French industrial and tertiary sectors

**DOI:** 10.1038/s41597-023-02542-z

**Published:** 2023-10-09

**Authors:** Kevin Bellinguer, Robin Girard, Alexis Bocquet, Antoine Chevalier

**Affiliations:** 1 MINES Paris, PSL University, Centre PERSEE - Centre for Processes, Renewable Energies and Energy Systems, Sophia Antipolis, 06904 Paris, France; 2 Technical Direction, Enedis, Courbevoie, 92400 France

**Keywords:** Energy modelling, Electrical and electronic engineering

## Abstract

The combination of ongoing urban expansion and electrification of uses challenges the power grid. In such a context, information regarding customers’ consumption is vital to assess the expected load at strategic nodes over time, and to guide power system planning strategies. Comprehensive household consumption databases are widely available today thanks to the roll-out of smart meters, while the consumption of tertiary premises is seldom shared mainly due to privacy concerns. To fill this gap, the French main distribution system operator, Enedis, commissioned Mines Paris to derive load profiles of industrial and tertiary sectors for its prospective tools. The ELMAS dataset is an open dataset of 18 electricity load profiles derived from hourly consumption time series collected continuously over one year from a total of 55,730 customers. These customers are divided into 424 fields of activity, and three levels of capacity subscription. A clustering approach is employed to gather activities sharing similar temporal patterns, before averaging the associated time series to ensure anonymity.

## Background & Summary

Today, the power network is confronted with rapid changes in the way we produce and consume electricity. The variability induced by increased consumption due to the roll-out of electric vehicles coupled with industry electrification is likely to put pressure on grid assets and generate expensive reinforcement strategies at critical locations on the grid. To cope with these issues, it is crucial to precisely assess the electricity demand from the consumer side with a fine temporal resolution.

For this purpose, and to comply with EU energy market legislation, Member States have deployed smart metering solutions at the residential level^1^ that precisely monitor household consumption. This promotes the growth of open source datasets dedicated to whole-house and domestic-appliance-level electricity demand. Interested readers may refer to former works^2,3^, which in addition to the introduction of their own datasets, provide summaries of available datasets at the time of writing. More recently, within the framework of the WPuQ^4^ research project, measurements were conducted from 2018 to 2020 in 38 German households. Usage-specific datasets are also found in the literature (e.g. electric vehicles^5^, heat pumps^4^).

While the scientific community tends to focus on residential demand, very little attention is paid to the tertiary sector. Typically, customers fall within several main categories of activity, including residential, commercial, industrial, and agricultural. In this work, the term “industrial and tertiary” should be understood as the complement to the residential sector that gathers not only tertiary activities (e.g. offices, administration, and education), but also primary and secondary businesses (e.g. farming, construction, heavy industry). Industrial and tertiary activities constitute a high electricity consumer that represented 64% of the French total consumption in 2019^6^. Despite the prevalence of this sector, a limited number of consumption datasets is available. This lack may be explained by the association of demand patterns with crucial and strategic production processes. Table 1 highlights that the literature dedicated to this field differs from that associated with the residential sector. Typically the former is built from a large number of facilities but at the cost of a coarse temporal granularity. Collection methods are also different; the industrial and tertiary sectors rely heavily on surveys and energy bills. A lack of French datasets is also noted. To fill these gaps, we introduce the Electrical Load Measurements Aggregated by business Sectors in France (ELMAS) dataset, a set of hourly load profiles dedicated to the industrial and tertiary sectors and derived from more than 55,000 companies.

**Table 1:** Open access electrical load datasets.

Name	Sector	Location	Duration	Collection methods	Temporal resolution	No. units
RECS^15^	Residential	US	1978 -	Collected from energy suppliers (energy bills)	Yearly consumption	18,496 (last survey)
REFIT^2^	Residential	UK	2-year long	Smart metering	8-s load time series	20
^4^	Residential	DE	May 2018 to the end of 2020	Smart metering	10-s to 1-h load time series	38
^16^	Residential	UR	Some weeks long to some years long	Smart metering	1- to 15-min load time series	110,953 (Agg. load)
UK-DALE^17^	Residential	UK	655 days (2012-2015)	Smart metering	16 kHz (whole-house), 1/6 Hz (individual appliances)	5
CBECS ^13,14^	Commercial	US	1979 -	Collected from energy suppliers (energy bills)	Yearly consumption	6,436 (last survey)
CEUS^30^	Commercial	CA	2018 - 2022	Survey performed by professionals	Yearly consumption and hourly load profiles	27,000 (expected)
^31^	Commercial	US	One year	Simulated from 16 reference buildings models^18^	Hourly, daily, and weekly load profiles for 16 climate zones	16 × 935
CoSSMic ^32^	Residential and small businesses	DE	2014-12-11 - 2019-05-01	Smart metering	Detailed household load per minute to hourly resolution	11
BPD^33,34^	Residential and commercial	US	2013 -	Online survey	Yearly consumption	>1,000,000
EULP ^35^	Residential and commercial	US	One year	Models calibrated from CBECS and RECS	15-min load time series	NA
^36^	Industrial and tertiary	DE	Two years (2016 or 2017)		15-min load time series	50
JERICHO-E-usage^37^	Residential, industrial, commercial, and mobility	DE	One year (2019)	Simulated from various sources (e.g. measured load profiles)	Hourly time series for 38 spatial regions	NA
ELMAS^20^	Industrial and tertiary	FR	One year (2018)	Smart metering	18 hourly load profiles	55,730

Agg. = Aggregate, US = United States, UK = United Kingdom, DK = Denmark, FR = France, DE = Germany, UR = Uruguay, CA = California. BPD, CBECS, RECS are periodic studies that accumulate collected information. The number of units for BPD represents the sum of all collected information, while for CBECS, and RECS, it represents the number of answers from the last survey.

**Fig. 1:**
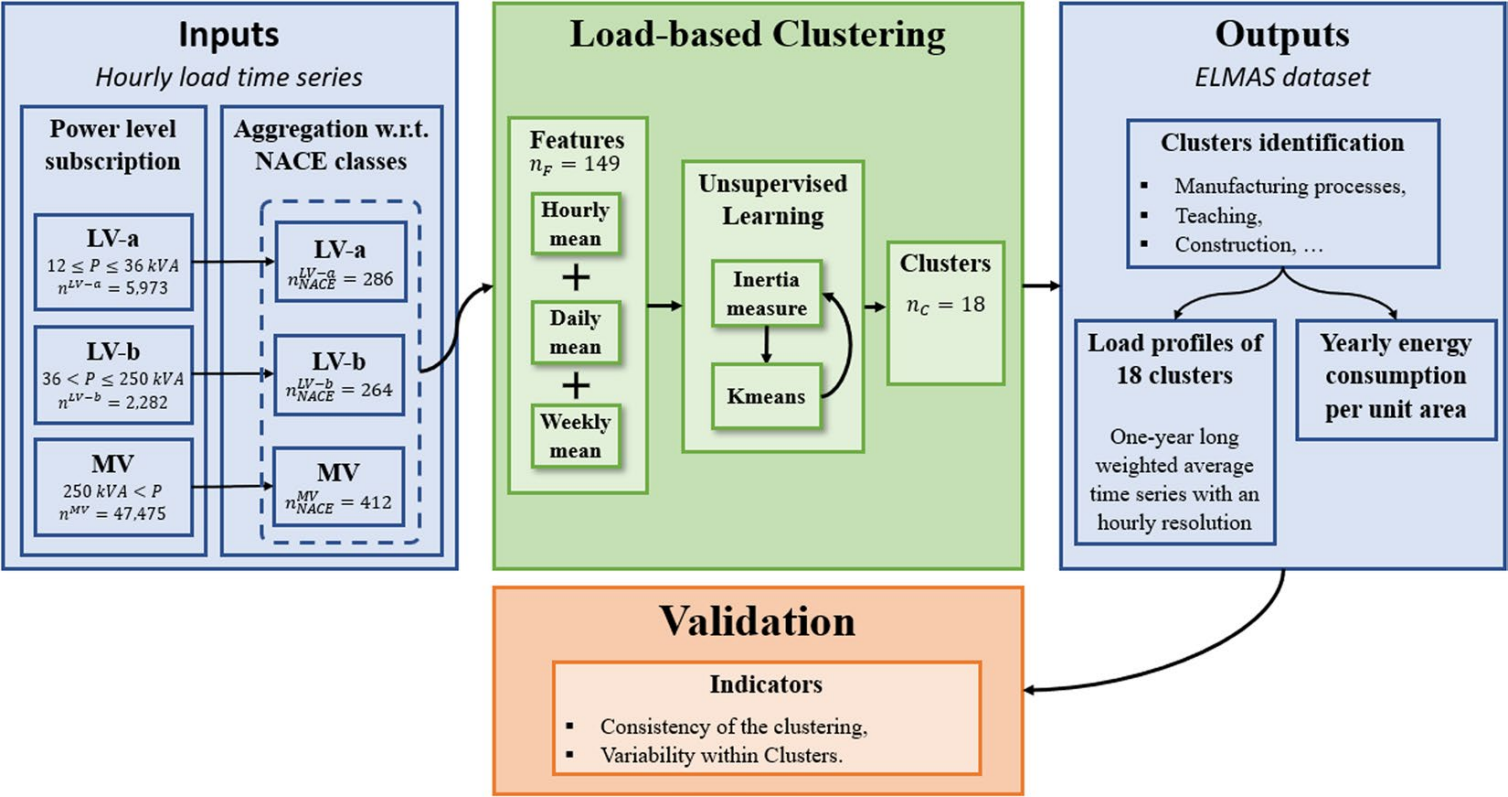
Overview of the load profile generation methodology. Inputs are composed of hourly load time series from 55,730 customers grouped into 424 business sectors, and three levels of subscribed power. A k-means clustering model based on temporal features is then used to derive groups of business sectors sharing similar consumption patterns. From these groups, generic load profiles are generated and validated.

Figure 1 provides an overview of the methodology used to derive the ELMAS datasets from hourly load measurements classified according to each customer’s subscribed capacity and business group. The customer’s field of activity follows the Statistical Classification of Economic Activities in the European Community (NACE)^7^ framework, which is a four-digit industry standard classification composed of 21 sections, 88 divisions, 272 groups, and 615 classes. This classification is an appealing approach to generate average load profiles w.r.t. fields of activity. Nevertheless, discrepancies between the temporal patterns of customers that belong to the same NACE section highlight the need to resort to another clustering approach. Thus, a K-means clustering algorithm is used to gather 424 business groups sharing similar temporal patterns into 18 clusters. An analysis of the main activities present in the clusters leads to their identification. Then, load profiles with an hourly resolution are generated. In addition to the consumption time series of these 424 business groups, we also have at our disposal the annual energy consumption of millions of customers. Such information makes it possible to develop weighted averaged load profiles that reflect the distribution of the various fields of activity at the national level.

This study contributes to the scientific literature by proposing numerical load profiles of a wide range of industrial and tertiary actors ranging from wholesale to agriculture. These profiles provide a better understanding of consumer behaviours at various temporal aggregation levels (that range from daily to weekly) thanks to their hourly resolution. In addition, the ELMAS dataset significantly stands out from other open access load datasets in the way data is recorded. Typically, scientific studies access a very limited panel of engaged customers, while in this paper, the French Distribution System Operator (DSO) provides us a set of nationally-distributed measurements thanks to the deployment of smart metering devices. To the authors’ knowledge, this is the first dataset that originates from a DSO database, which makes it unique and valuable. This collaboration makes it possible to supply load profiles related to very specific fields of activities seldom found in the literature (e.g. food industries, property management companies). As these load profiles may be associated with strategic industrial processes, and their disclosure may negatively impact the stakeholders, it is necessary to preserve the anonymity of customers. To this end, inputs data are not shared, all the more as they follow the General Data Protection Regulation (GDPR) framework, and outputs data, namely load profiles, are provided at a level of aggregation that prevents any identification. This is the first dataset that represents the demand of both industrial and tertiary sectors in France, and with a finer temporal resolution than the monthly energy bills typically used in other countries.

The proposed profiles are of great interest to guide medium- to long-term power system planning (e.g. to identify actionable demand drivers^8^), and to evaluate the consumption trajectory of a sector (e.g. to assess the impacts of energy efficiency measures^9^, technological developments, or to evaluate the demand-side flexibility potential^10^). There is no doubt that stakeholders such as urban planners and electricity retailers will find interest in this source of information in the frame of energy modelling strategies. The ELMAS dataset can populate the bottom-up energy model of an urban area to determine the expected load profile at any point in the network. In that sense, it contributes to guiding investment road maps. The proposed dataset can also be used to calibrate parameters of bottom-up models such as MOSAIC^11^ or FORECAST^12^.

## Methods

In the scientific literature, it is challenging to access the electricity consumption records of industrial and tertiary companies due to confidentiality issues. Here, we propose generic electricity consumption profiles associated with 18 relevant business sectors (e.g. trade, education) derived from 55, 730 consumption time series initially split into 424 business sectors and three levels of subscribed capacity. To preserve anonymity, a two-level clustering approach is employed. First, the time series of the various companies are aggregated w.r.t. to their business sectors and their subscribed level of power. Then, a clustering approach is performed on standardised time series to group business sectors that share similar temporal patterns, before aggregating them.

### Data measurements

#### Electricity consumption

Energy consumption data for buildings originates from different sources. Databases can be collected from surveys of energy suppliers, respondents, and even from utility bills. In such cases, data typically have a monthly resolution^13–15^. The retrieval of data can also be automated through the use of smart meters^2,4,16,17^, which provide information at a lower time resolution. Databases can also be generated from simulation models that mimic the building occupiers’ behaviour. In this regard, the US department of energy has created commercial reference building models^18^ which are composed of 16 building types.

In this study, load data is initially collected through Linky^19^ digital meters at the building level by Enedis, the main French DSO. This building-level dataset does not provide information regarding the energy use of appliances and equipment. In total, the hourly time series from 55, 730 industrial and tertiary companies are gathered over the year 2018. This year is divided into 52 weeks starting from January. Special attention has been paid by Enedis to selecting companies with at least one year of consumption measurements and with a high degree of data integrity (i.e. observations that do not mimic an effective consumption behaviour are rejected).

**Fig. 2:**
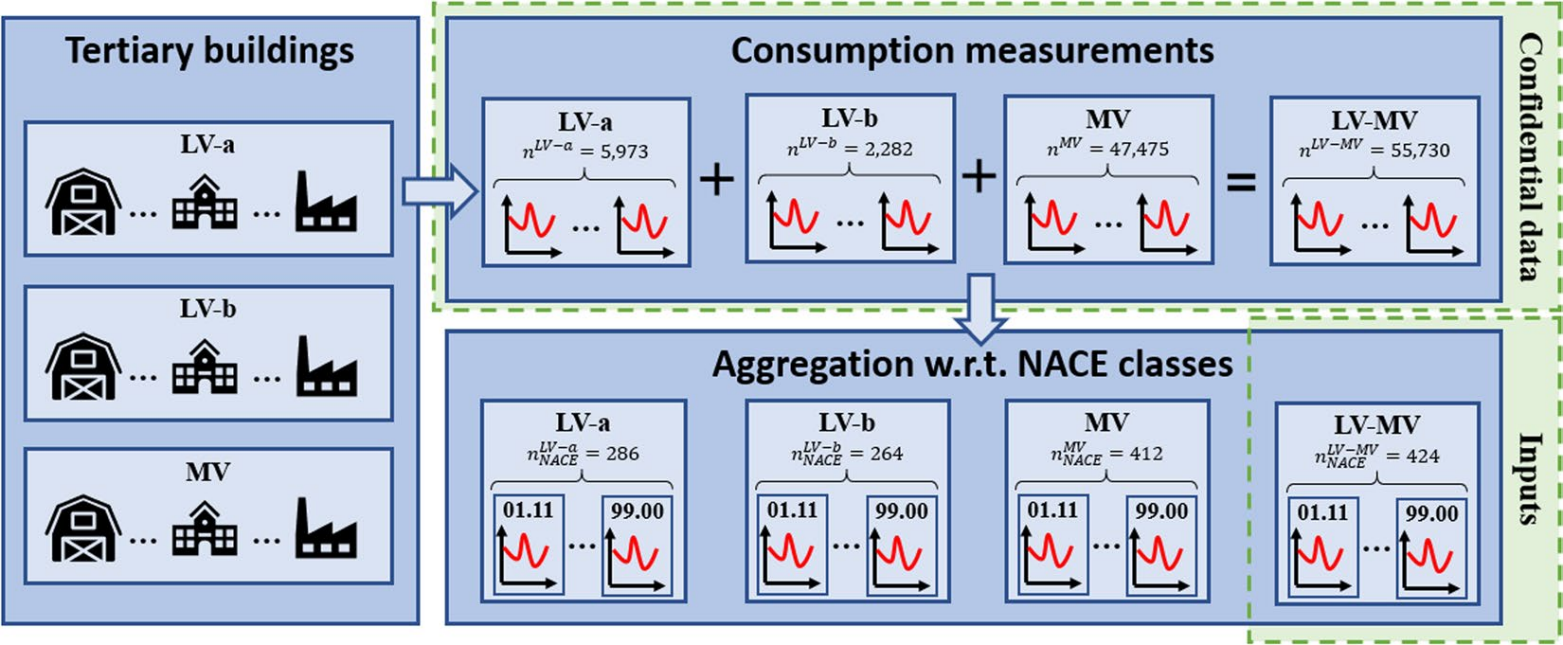
Overview of the generation process of aggregated data used as inputs in the clustering-based approach. The consumption measurements at the company level are aggregated according to the NACE classes for privacy reasons. The *LV-a*, *LV-b*, and *MV* levels respectively possess 286, 264, and 412 NACE classes. The combination of these three levels allows us to fill mutual gaps in terms of NACE classes, reaching a total of 424 NACE classes. In this study, only aggregated data from the *LV-MV* group are investigated.

Consumption time series are gathered into three levels of subscribed power: (1) the *LV-a* segment gathers customers connected to the low-voltage network that have subscribed to power between 12 and 36 kVA, (2) the *LV-b* class corresponds to customers connected to the low-voltage network with a subscribed capacity ranging from 36 to 250 kVA, and (3) the *MV* class represents customers connected to the high-voltage network with a power subscription greater than 250 kVA. Concurrently, industrial and tertiary consumers are also grouped according to their NACE coding, which is a statistical classification of economic activities used at the European level and more specifically in France. In this study, we focus on two levels of heading of the NACE structure; namely the 21 sections identified by alphabetical letters A to U, and 424 out of the 615 available classes identified by four-digit numerical codes (01.11 to 99.00). For the reader’s convenience, Table 2 provides a brief description of some of the classes associated with the 21 sections, while a complete description is given in the file NACE_classification.csv^20^. For confidentiality reasons, sensitive information regarding customers (e.g. name, location) are not disclosed by Enedis. To the same end, consumption time series are aggregated according to the NACE classification (Fig. 2).

**Table 2:** Brief description of some of the 424 classes used in this study. A detailed list of all the NACE classes used in work is proposed in the document NACE_classification.csv^20^. For the complete NACE classification, interested readers may refer to^38^.

Sections	Classes
(A) Agriculture, forestry and fishing	(01.11) Growing of cereals (except rice), leguminous crops and oil seeds / (01.12) Growing of rice / (01.13) Growing of vegetables and melons, roots and tubers, etc.
(B) Mining and quarrying	(5) Mining of coal and lignite / (6) Extraction of crude petroleum and natural gas / (7) Mining of metal ores, etc.
(C) Manufacturing	(10.1) Processing and preserving of meat and production of meat products / (10.20) Processing and preserving of fish, crustaceans and molluscs / (10.3) Processing and preserving of fruit and vegetables, etc.
(D) Electricity, gas, steam and air conditioning supply	(35) Electricity, gas, steam and air conditioning supply.
(E) Water supply, sewerage, waste management and remediation activities	(36.00) Water collection, treatment and supply / (38) Waste collection, treatment and disposal activities; materials recovery.
(F) Construction	(41.10) Development of building projects / (41.20) Construction of residential and non-residential buildings / (42.11) Construction of roads and motorways, etc.
(G) Wholesale and retail trade; repair of motor vehicles and motorcycles	(45.11) Sale of cars and light motor vehicles / (45.19) Sale of other motor vehicles / (45.20) Maintenance and repair of motor vehicles, etc.
(H) Transportation and storage	(49.10) Passenger rail transport, interurban / (49.20) Freight rail transport / (49.31) Urban and suburban passenger land transport, etc.
(I) Accommodation and food service activities	(55.10) Hotels and similar accommodation / (55.20) Holiday and other short-stay accommodation / (55.30) Camping grounds, recreational vehicle parks and trailer parks, etc.
(J) Information and communication	(58.11) Book publishing / (58.12) Publishing of directories and mailing lists / (58.13) Publishing of newspapers, etc.
(K) Financial and insurance activities	(64.11) Central banking / (64.19) Other monetary intermediation / (64.20) Activities of holding companies, etc.
(L) Real estate activities	(68.10) Buying and selling of own real estate / (68.20) Rental and operating of own or leased real estate / (68.31) Real estate agencies, etc.
(M) Professional, scientific and technical activities	(69.10) Legal activities / (69.20) Accounting, bookkeeping and auditing activities; tax consultancy / (70.10) Activities of head offices, etc.
(N) Administrative and support service activities	(77.11) Rental and leasing of cars and light motor vehicles / (77.12) Rental and leasing of trucks / (77.22) Rental of video tapes and disks, etc.
(O) Public administration and defence; compulsory social security	(84.11) General public administration activities / (84.12) Regulation of the activities of providing health care, education, cultural services and other social services, excluding social security / (84.13) Regulation of and contribution to more efficient operation of businesses, etc.
(P) Education	(85.10) Pre-primary education / (85.20) Primary education / (85.31) General secondary education, etc.
(Q) Human health and social work activities	(86.10) Hospital activities / (86.21) General medical practice activities / (86.22) Specialist medical practice activities, etc.
(R) Arts, entertainment and recreation	(90.01) Performing arts / (90.02) Support activities to performing arts / (90.03) Artistic creation, etc.
(S) Other service activities	(94.11) Activities of business and employers membership organisations / (94.12) Activities of professional membership organisations / (94.20) Activities of trade unions, etc.
(T) Activities of households as employers; undifferentiated goods- and services-producing activities of households for own use	(97.00) Activities of households as employers of domestic personnel / (98.20) Undifferentiated service-producing activities of private households for own use, etc.
(U) Activities of extraterritorial organisations and bodies	(99.00) Activities of extraterritorial organisations and bodies.

**Fig. 3:**
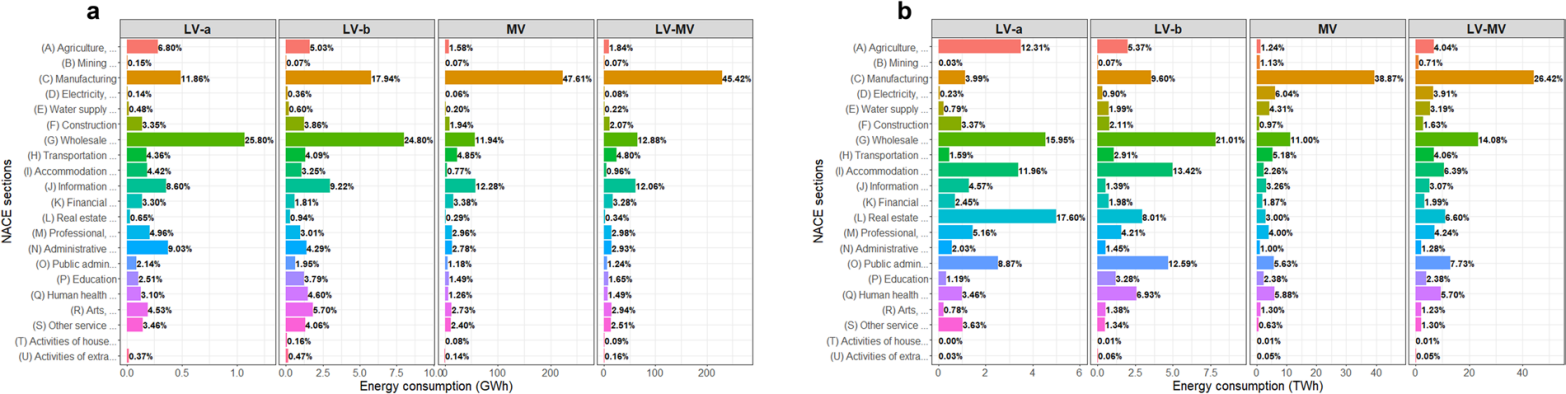
Distributions of the annual energy consumption according to the NACE sections and the subscribed level of power. Special attention should be paid to the different order of magnitude between the three capacity levels. The percentages represent the proportion of energy consumption for the considered customers segment. The files Annual_energy_time_series.csv and Annual_energy_weights.csv^20^ respectively gather the numeric values used to generate these graphs. (**a**) This data is derived from the set of 55,730 customers that provides hourly consumption time series. This set constitutes the main input to generate the load profiles of the ELMAS dataset. (**b**) This data is derived from a larger panel of around 4,534,891 customers that provides annual energy consumption. This source of information is used to correct sampling bias of the former set.

In the next steps, load consumption time series from the three customer segmentation levels are considered simultaneously to fill gaps in terms of missing NACE classes, and are denoted as the *LV-MV* group. Indeed, the *LV-a* and *LV-b* groups contain respectively 286 and 264 classes, while the *MV* set comprises 412 classes. In total, we have at our disposal 424 classes, some of which contain several load time series associated with distinct groups of subscribed capacity. This new group is characterised by predominant NACE sections in terms of annual energy consumption (Fig. 3): examples include the *(C) Manufacturing*, *(G) Wholesale and retail trade*, *(O) Public administration and defence*, and *(P) Education* sections.

#### Annual energy consumption and surface area

The energy consumption time series dataset represents a limited panel composed of 55, 730 customers, which may bias the output load profiles in comparison with the whole French panel of industrial and tertiary customers. To fill this gap, Enedis provides the annual energy consumption of a wider range of customers for the year 2019. Thus, we have at our disposal the annual energy consumption of 4, 030, 708 customers for the *LV-a* segment, 408, 183 clients for the *LV-b* class, and around 96, 000 customers for the *MV* group. The aggregated energy consumption of each NACE class (Fig. 3) is employed in the weighting strategy of the clustering approach to reflect national tendencies. In addition, the DSO also provides the surface area of buildings that belong to the *LV-a* / *LV-b* customer segmentation. This database, which associates surface area and annual energy consumption, is composed of 994, 790 customers gathered into 426 NACE classes.

#### Weather data

External factors such as the weather may have a significant impact on the load consumption. For instance, temperature highly influences the load consumption of buildings equipped with electric heaters and air conditioners. This dependency may be characterised by the thermosensitivity parameter, which measures the variation of the electric consumption w.r.t. the variation of the outdoor temperature. This criterion is used during the validation stage to measure the homogeneity of the derived load profiles. In this study, we consider measurements from Meteo-France, the French national meteorological service, at 32 main cities spatially distributed in France. Then, a weighted average aggregates these observations at the national level. The weights are proportional to the energy consumption dedicated to thermal uses (i.e. electric heating, air conditioning). Thus, regions associated with higher thermosensitivity are more represented in the computation of the temperature. The resulting time series are provided in the file Temperature.csv^20^.

### Electricity load curve profiling

Load profiling consists in generating consumption patterns for a given customer over a defined period of time. Wang *et al*. provide fairly a complete review regarding load profiling^21^. This process can be divided into five stages: (1) load data preparation, (2) load curve clustering, (3) clustering evaluation, (4) customer segmentation, and (5) result application. Therefore, clustering is the core technique of load profiling: it segregates consumption time series sharing similar patterns in the same cluster, while different clusters gather diversified information. From these clusters typical load curves are then derived.

#### Data pre-processing

The dataset under study is composed of variables of comparable units but with various magnitude and variances. The purpose of this paper is to gather data exhibiting similar temporal patterns rather than similar levels of magnitude. It is good practice to normalise or standardise input data in the frame of data clustering so that large-scale or high-variance features do not dominate the results. Thus, all of the time series are standardised following Equation (1), which implies that the resulting time series have zero-mean and unit-variance.

1 $$\overline{{X}_{i}}=\frac{{X}_{i}-{\mu }_{i}}{{\sigma }_{i}}$$

$$\begin{array}{l}\overline{{X}_{i}}\,\,\,\mathrm{Standardised\ load\ profile\ for\ NACE\ class}\,\,i\,[{\rm{\varnothing }}],\\ {\mu }_{i}\,\,\,\mathrm{Mean\ energy\ consumption\ throughout\ the\ year\ of\ class}\,\,i\,[kWh],\\ {\sigma }_{i}\,\,\,\mathrm{Standard\ deviation\ of\ the\ time\ series}\,\,i\,[kWh].\end{array}$$

#### Feature space

**Fig. 4:**
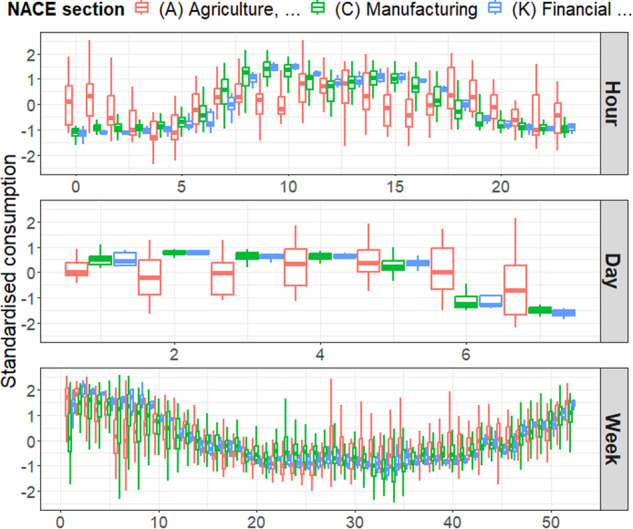
Distributions of the averaged hourly standardised consumption of three NACE sections according to the hour of the day, the day of the week, and the week of the year for the *LV-a* level. The *A*, *C*, and *K* sections respectively contain 18, 40, and 9 classes.

At this point, data clustering based on the NACE sections can be viewed as an easy and straightforward option to generate load profiles. Nevertheless, we observe through Fig. 4 that some sections, such as section *(A) Agriculture, forestry and fishing*, exhibit a wide intra-cluster variability for the three temporal resolutions considered. In addition, this variability may evolve over time. For instance, companies associated with section *(C) Manufacturing* display similar consumption behaviour during nighttime, while significant differences are observed during daytime. On the contrary, other economic activities, such as those related to section *(K) Financial and insurance activities*, behave similarly.

**Fig. 5:**
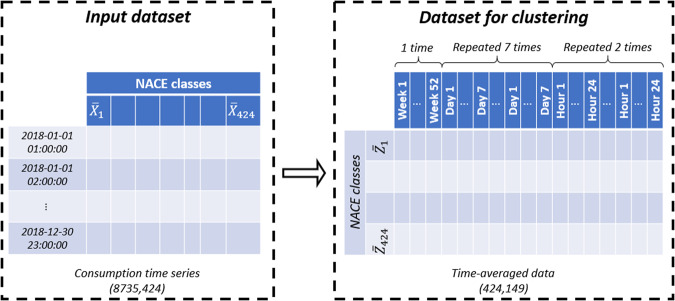
Structure of the features space used for the clustering step.

As a result, clustering according to NACE section is not relevant regarding consumption patterns. This motivates us to opt for an alternative clustering approach based on temporal patterns. Consumption time series are then processed to build the features space in which the clustering algorithm is run. This space is composed of the hourly, daily, and weekly averaged consumption for each NACE class (Fig. 5). Hourly and daily data are repeated respectively 2 and 7 times to avoid an over-representation of weekly measurements. The newly created features are designated by the variable $\overline{Z}$. Such a space enables us to identify NACE classes that share similar consumption patterns on an hourly, daily, and weekly basis.

#### Clustering approach

##### Model

The literature proposes several definitions of clusters that lead to the development of specific algorithms (e.g. distance- or density-based algorithms). Thus, a plethora of clustering techniques are developed^22^, and applied in a wide range of fields that range from renewable energy production forecasting^23^ to disease diagnosis^24^. In this study we consider the K-means algorithm^25^, which is probably one of the most frequently used algorithms for clustering data due to its simplicity and ability to reach near-optimal solutions quickly. In short, the K-means algorithm is a partitional algorithm that minimises the distance between points in a cluster with the point designated as the centre of that cluster. That centre of the mass, or centroid, may not necessarily belong to the dataset. As an unsupervised learning machine algorithm, it does not require any prior knowledge about the dataset, except an a priori number of clusters, *c*, defined by the user.

The creation and definition of clusters is performed as follows. First during the initialisation step, the algorithm randomly chooses *c* features from the set ${{{\mathcal{Z}}}}=\left\{{\overline{Z}}_{1},\cdots \,,{\overline{Z}}_{424}\right\}$, which gathers the temporal characteristics of the 424 NACE classes. These *c* NACE classes are used as initial centroids, and constitute the set ${{{\mathcal{A}}}}=\left\{{\overline{A}}_{1},\cdots \,,{\overline{A}}_{c}\right\}$. Then, a sequence of two steps is repeated until a stopping criterion is met (e.g. the maximum iteration threshold is reached or no change in cluster assignment is observed).

First, during the assignment step, each NACE class, *j*, is assigned to the nearest cluster by minimising an objective function (Equation (2)) based on the Euclidian distance metric^26^. A weighting strategy that considers the annual electricity consumption of the NACE class is adopted to account for discrepancies in energy consumption between the different classes. Therefore, more importance is given to classes associated with higher levels of energy consumption. The annual energy consumption of each NACE class is provided in the file Annual_energy_weights.csv^20^.

2 $$\begin{array}{lll}J({\overline{Z}}_{j},{{{\mathcal{A}}}}) & = & \mathop{\sum }\limits_{k=1}^{c}{z}_{jk}{\omega }_{j}{\parallel {\overline{Z}}_{j}-{\overline{A}}_{k}\parallel }^{2},\,\mathrm{with}\\ {z}_{jk} & = & \left\{\begin{array}{ll}1 & \,\mathrm{if}\,{\parallel {\overline{Z}}_{j}-{\overline{A}}_{k}\parallel }^{2}={\min }_{1\le g\le c}{\parallel {\overline{Z}}_{j}-{\overline{A}}_{g}\parallel }^{2}\\ 0 & \,\mathrm{otherwise.}\end{array}\right.,\mathrm{and}\\ {\omega }_{j} & = & \frac{{E}_{j}}{\mathop{\sum }\limits_{l=1}^{{N}_{NACE}}{E}_{l}}\end{array}$$

$$\begin{array}{l}{\overline{Z}}_{j}\,\,\,\mathrm{Temporal\ features\ of\ the\ NACE\ class}\,\,j,\\ {\overline{A}}_{k}\,\,\,\mathrm{Temporal\ features\ associated\ with\ the\ centroid}\,\,k,\\ {z}_{jk}\,\,\,\mathrm{A\ binary\ variable\ indicating\ if\ the\ data\ point}\,{\overline{Z}}_{j}\,\,\mathrm{belongs\ to\ the}\,\,{k}^{th}\,\mathrm{cluster},\\ {\omega }_{j}\,\,\,\mathrm{Weight\ associated\ with\ the\ NACE\ class}\,\,j,\\ {E}_{j}\,\,\,\mathrm{Annual\ energy\ consumption\ of\ NACE\ class}\,\,j,\\ {N}_{NACE}\,\,\,\mathrm{Number\ of\ NACE\ classes}\,\,(\,\mathrm{here},{N}_{NACE}=424).\end{array}$$

After all the points are assigned, the second step consists in updating the centroids’ positions following Equation (3). During this updating step, the centroids are recalculated as the weighted average of all data points assigned to a specific cluster.

3 $${\overline{A}}_{k}=\frac{\mathop{\sum }\limits_{i=1}^{n}{z}_{ik}{\omega }_{i}{\overline{Z}}_{i}}{\mathop{\sum }\limits_{i=1}^{n}{z}_{ik}{\omega }_{i}}$$

K-means results are sensitive to the initial cluster centres (i.e. generated during the initialisation step), which is why the algorithm is usually run several times. Here the clustering model is run 5 times, then the final clusters are generated with the averaged of the previously determined clusters centre as starting points.

##### Quality of the clusters

The K-means algorithm requires the user to define the number of clusters *c* to perform data clustering. However, this value is usually unknown for real applications. Several approaches are developed in the literature to address this issue. Typically, *a posteriori* approaches are employed: the quality of the clustering structure is assessed for several numbers of clusters after the algorithm is run. A good clustering can be defined as a structure characterised by compact and well-separated clusters. Compactness refers to the closeness of the samples to the centroids, in other words it means than samples are similar, while separation denotes that different clusters carry distinct information (visually the clusters do not overlap in the feature space). In this work, three intrinsic methods are considered to assess the quality of the clustering, namely, distortion, inertia, and silhouette scores:

1. The distortion score computes the average of the squared distances from the cluster centres of the respective clusters. Therefore, the closer the data points are to the centroid of the cluster, the lower the distortion. In other words, tight clusters are associated with a low distortion score.
2. Inertia is derived from the within cluster sum of squares: for each cluster, we compute the weighted squared distance between all the points of this cluster and the centroid, and then sum up the distances. Therefore, a small inertia value indicates a coherent set of clusters.
3. The silhouette index^27^ assesses the cohesion and separation of clusters, which means that a good score is reached when clusters are tight and far from each other. This measure, which is performed for every sample and ranges from − 1 to + 1, indicates how well the point lies within its cluster, and poorly matches neighbouring clusters. A silhouette coefficient close to 1 / 0 / − 1 respectively means that the data point is far from the neighbouring clusters / close to the decision boundary / or may be assigned to the wrong cluster. The graphical display associated with the silhouette coefficients offers a synthetic view of the quality of the clusters for the entire sample. In order to obtain an overview, we compute the mean silhouette coefficient of all samples for different numbers of clusters. Therefore, we are seeking the clustering configuration that leads to the highest mean silhouette value.

**Fig. 6:**
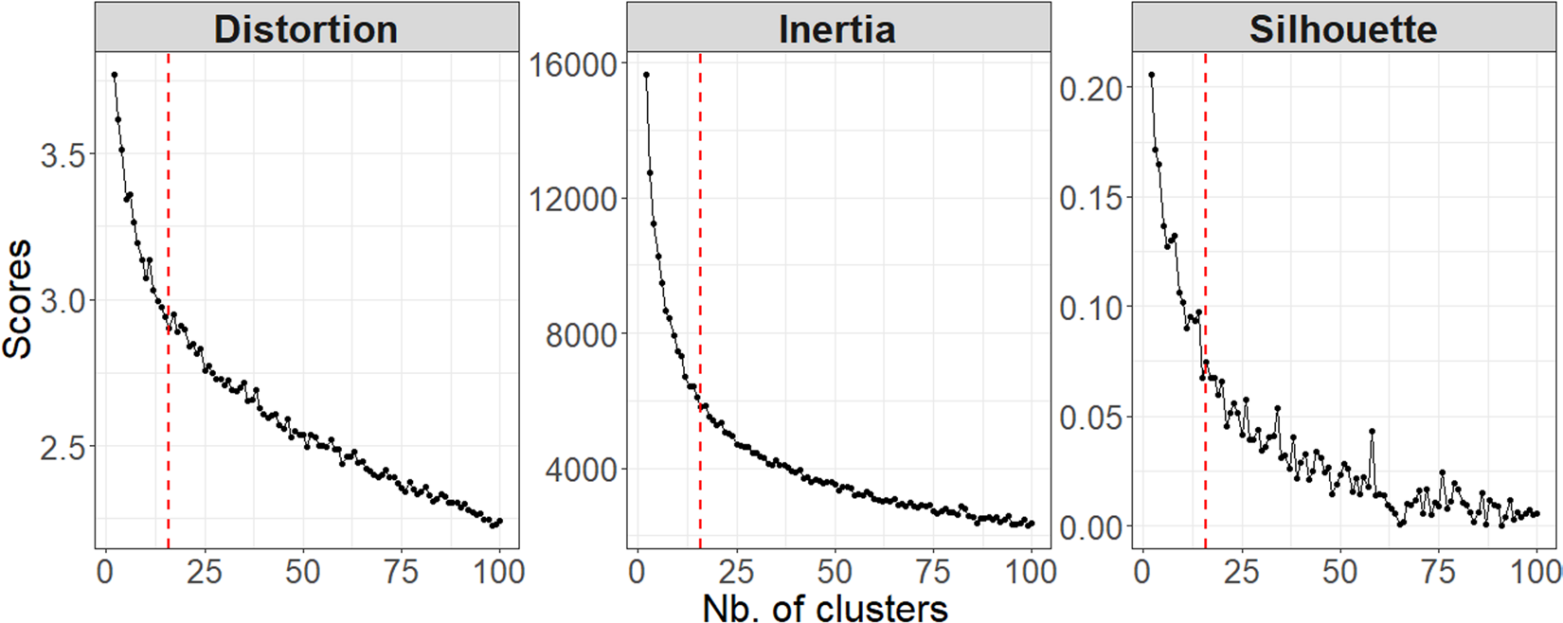
Distortion, inertia, and silhouette curves against the number of clusters used with the K-means algorithm. The red dashed line represents the identified elbow point (here *c* = 16).

As the complexity (i.e. the number of clusters) increases, so does the coherence of the clustering; a trade-off has to be found between maximising the quality of the clustering, and minimising the complexity of the model. To find the optimal number of clusters, the elbow method is usually chosen. Such a tool is based on the graphical representation of the quality scores. This consists in finding the number of clusters after which the decrease in distortion/inertia begins to slow down. In other words, the “elbow” point represents the number of clusters from which the increase in the number of clusters has little effect on the scores. The main drawbacks of this approach are that it relies on a subjective identification of the elbow, and requires running the clustering model for a large range of clusters. In Fig. 6 one can identify the elbow of the distortion and inertia curves at the 16^th^ cluster. At this identified point, the mean silhouette value remains acceptable.

#### Misclassification

**Fig. 7:**
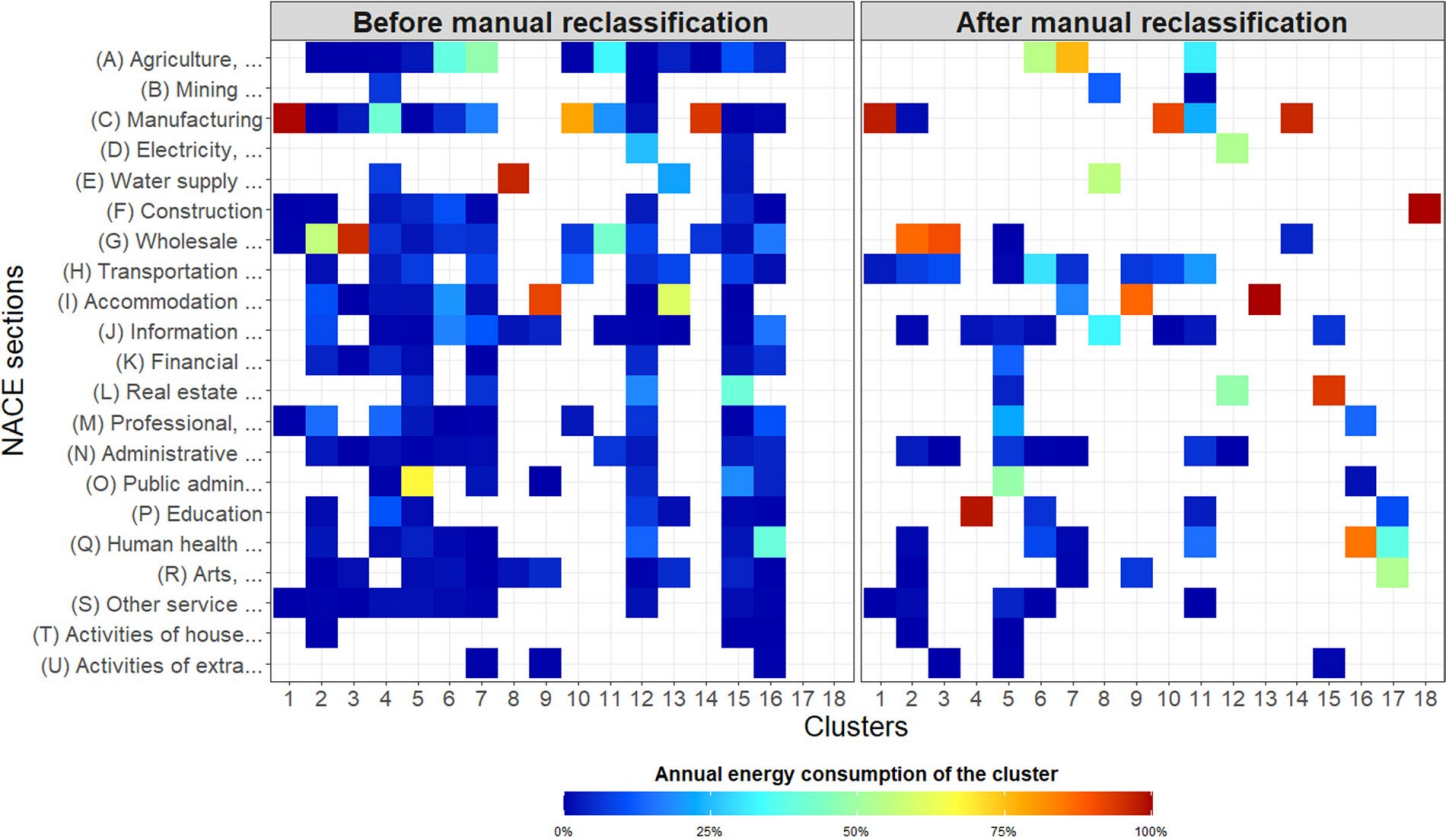
Distribution of the NACE sections in the clusters before and after the manual reclassification. The colours stand for the annual energy consumption of the cluster.

A thorough analysis of the clusters derived reveals that they are typically dominated by some NACE sections; for instance Fig. 7 shows that the greatest share of the annual energy consumption of cluster 1 is due to the NACE section *(C) Manufacturing*. However, numerous NACE sections are scattered over various clusters, which increases the global heterogeneity of the clustering while spoiling the interpretation of the clustered data. The proportion of these dispersed NACE classes in terms of annual energy consumption remains low, which suggests that a manual reorganisation has little impact on the global consistency of the clusters. This manual reclassification is conducted in such a way that scattered NACE classes are gathered in the cluster that possesses the highest share of the considered NACE section, while taking into account the specificity of the section. For instance, we note that the NACE section *(C) Manufacturing* is spread over 14 clusters. The main shares of this section are gathered in order of importance in clusters 1, 14, 10, and 4. Activities present in cluster 1 are mainly related to manufacturing processes, just like those classified in cluster 4, while activities in clusters 14 and 10 are respectively devoted to bakery and the wine industry. Therefore, NACE classes of clusters 1 and 4 are gathered within cluster 1. This process is repeated for all NACE sections. This reclassification step is partly automated through a search for specific wording. Thus, NACE classes that contain the word “office” are gathered in cluster 5. In addition, two new clusters are generated at the end of this manual reclassification; namely clusters 17 and 18, which gather respectively activities related to the arts, human health, and construction. The creation of these additional clusters originated from the need to provide clusters dedicated to specific fields of activity.

Interested readers can find a description of the clustering before (Cluster_before_manual_reclassificatio.csv^20^) and after this manual reclassification (Cluster_after_manual_reclassification.csv^20^). Hereafter, only the second version of the clustering is considered.

#### Generation of load profiles

The next step consists in deriving load profiles for the set of clusters obtained previously. To do so, data associated with the various NACE classes are averaged w.r.t. the cluster they belong to. A weighted average (Equation (4)) based on the annual energy consumption of the NACE classes is employed to account for the prevalence of high energy consumers. The weighted average of the 18 clusters is provided in the file Time_series_18_clusters.csv^20^.

4 $${\overline{Y}}_{j}=\frac{\sum _{k\in {C}_{j}}{\omega }_{k}\cdot {\overline{X}}_{k}}{\sum _{k\in {C}_{j}}{\omega }_{k}}$$

$$\begin{array}{l}{\overline{Y}}_{j}\,\,\,\mathrm{Standardised\ weighted\ average\ load\ profile\ for\ cluster}\,\,j\,[{\rm{\varnothing }}],\\ {C}_{j}\,\,\,\mathrm{Set\ of\ NACE\ classes\ that\ belong\ to\ cluster}\,\,j,\\ {\omega }_{k}\,\,\,\mathrm{Annual\ energy\ consumption\ of\ the\ NACE\ class}\,\,k\,[kWh],\\ {\overline{X}}_{k}\,\,\,\mathrm{Standardised\ time\ series\ of\ the\ NACE\ class}\,\,k\,[{\rm{\varnothing }}].\\ \end{array}$$

The identification process of the generated clusters as well as a detail analysis of their properties is provided in the supplementary material ELMAS_data_analysis.pdf^20^.

Finally, Table 3 provides the averaged annual energy consumption per unit area associated with the 18 identified clusters. This table is derived following Equation (5), and the annual energy consumption and surface of the 426 NACE sectors given in the file Energy_consumption_per_unit_surface_area.csv^20^. It is worth mentioning that the surface and annual energy consumption of 10 NACE classes are missing, namely the classes: 01.29, 84.22, 84.24, 97.00, 01.12, 01.15, 02.30, 17.11, 84.21, and 98.20. No imputation strategies have been investigated to fill these gaps.

5 $${E}_{j}^{S}=\frac{\sum _{k\in {C}_{j}}{E}_{k}}{\sum _{k\in {C}_{j}}{S}_{k}}$$

$$\begin{array}{l}{E}_{j}^{S}\,\,\,\mathrm{Annual\ energy\ consumption\ per\ unit\ area\ of\ cluster}\,\,j\,[\,\mathrm{kWh}/{m}^{2}],\\ {C}_{j}\,\,\,\mathrm{Set\ of\ NACE\ classes\ that\ belong\ to\ cluster}\,j\\ \,\,\,\,\mathrm{obtained\ with\ the\ K \mbox{-} means\ clustering\ approach},\\ {E}_{k}\,\,\,\mathrm{Annual\ energy\ consumption\ of\ the\ NACE\ class}\,\,k\,[kWh],\\ {S}_{k}\,\,\,\mathrm{Surface\ area\ of\ buildings\ that\ belong\ to\ the\ NACE\ class}\,\,k\,[{m}^{2}].\end{array}$$

**Table 3:** Annual electricity consumption per unit area of the 18 clusters. These values are derived from the consumption and surface area of customers that belong to the *LV-a* and *LV-b* segments.

Cluster ID	Cluster name	Consumption (kWh/m^2^)
1	Manufacturing process	40.86
2	Trades (non food)	56.09
3	Trades (food)	64.13
4	Education	40.01
5	Office	69.88
6	Crop farming and transportation	41.47
7	Livestock farming	5.01
8	Water supply and telecommunications	33.85
9	Restaurants	126.44
10	Food industry	90.27
11	Wine industry	73.37
12	Energy supply and rental activities	46.23
13	Hotels	63.27
14	Bakery	350.27
15	Property management companies	42.47
16	Hospital activities	91.70
17	Recreational and social activities	40.86
18	Construction	47.58

## Data Records

**Fig. 8:**
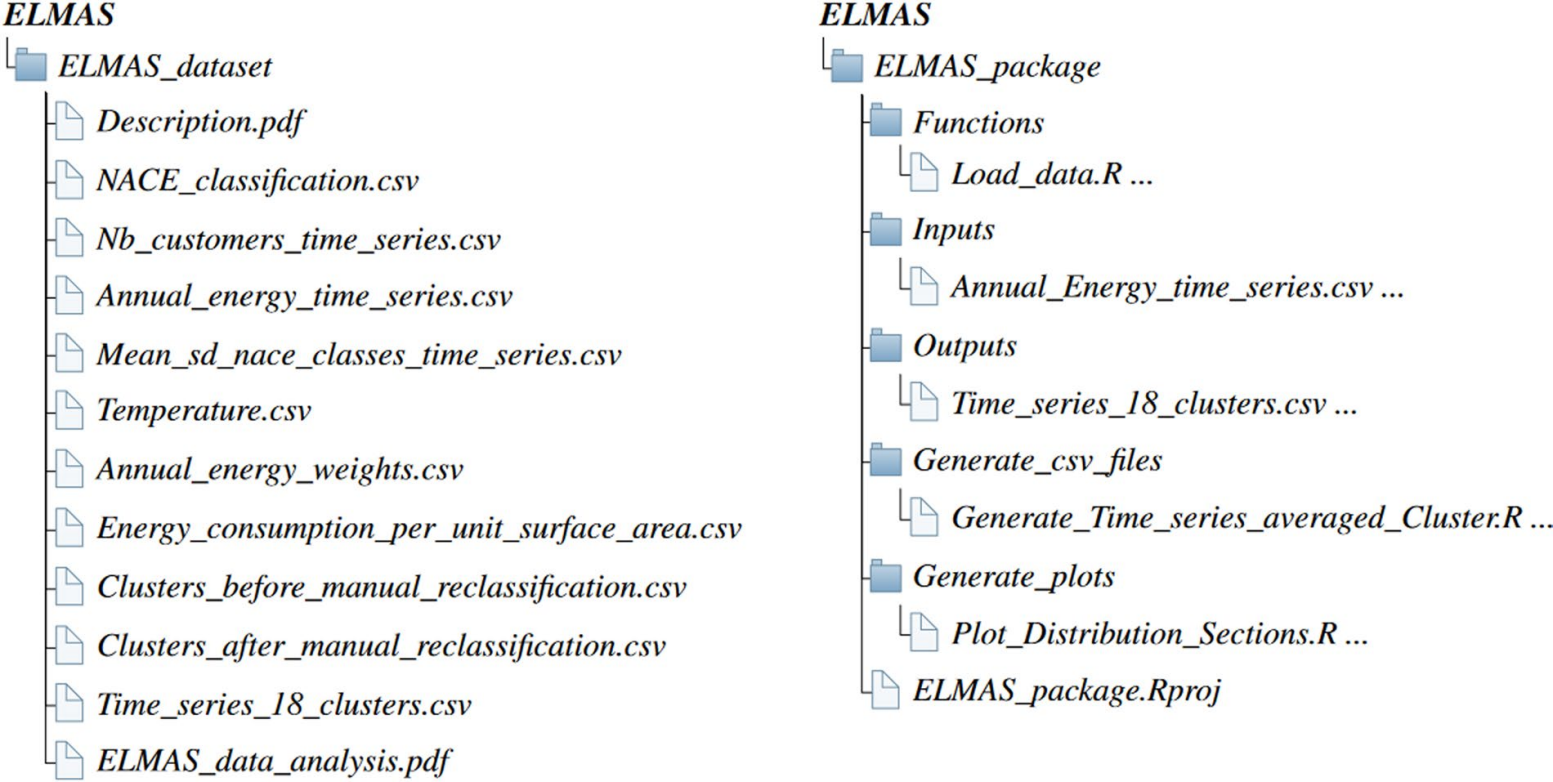
Directory structure of the ELMAS sub-folder. The ELMAS_dataset folder contains the data used to produce the plots of this paper, and the derived clusters. In addition, the documents Description.pdf and ELMAS_data_analysis.pdf respectively provide a description of the dataset and a detailed analysis of the derived clusters and load profiles. The ELMAS_package sub-folder gathers the R scripts used to generate the plots and some of the .csv files.

The raw data used in this project is collected and supplied by Enedis as part of a collaboration between the authors of this work. This source of information follows the General Data Protection Regulation^28^, as such it cannot be shared due to confidentiality restrictions. The first level to make the dataset anonymous consists in aggregating the consumption of industrial and tertiary companies that belong to the same NACE class. The resulting data constitutes the inputs of our approach. However, even at this level, some fields of activity can be identified because they exhibit specific load patterns. Under these circumstances, this dataset can not be shared publicly due to privacy concerns. Others wishing to repeat this work or perform similar studies should contact Enedis directly, and integrate them within a research project. Except this dataset, all data used in this work are available on the public repository, figshare^20^. The structure of the provided data is illustrated in Fig. 8: the *ELMAS_dataset* sub-folder gathers the datasets mentioned in the previous sections, while the *ELMAS_package* sub-folder collects the R script used to generate the data and the plots.

The *ELMAS_dataset* sub-folder contains two type of files, namely portable document format (.pdf) files that describe and analyse the numeric data provided as comma-separated value (.csv) files. The first row of .csv files indicates the name of the columns, while time data follows the French standard, namely: “DD/MM/YYYY hh:mm”. The first file of this sub-folder is a a description of the dataset structure (Description.pdf). Then, two batches of data can be distinguished: (1) information regarding the inputs used to derived the ELMAS database, and (2) data related to the outputs of the clustering approach. Hereinbelow, we detail the different csv files, while Table 4 describes the meaning of their columns. The first batch is composed of the description of NACE sections/classes and the associated coding (NACE_classification.csv). The files Nb_customer.csv and Annual_energy_time_series.csv gather respectively the number of customers and the annual energy consumption w.r.t. the NACE class and the level of subscribed power. The average consumption and the standard deviation associated with each NACE class is given in Mean_Sd_Nace_classes.csv. The file Temperature.csv contains the temperature time series of France. The weights (i.e. the annual energy consumption of the larger panel of customers) used to cluster the inputs, and to generate the weighted average time series of the clusters are given in Annual_energy_weights.csv, while the file Energy_consumption_ per_unit_surface_area.csv associates the annual energy consumption with the surface area of the building. The second batch of files is related to the data generated after the clustering. The files Cluster_before_manual_reclassification.csv and Cluster_after_manual_reclassification.csv assign a cluster to each NACE class before and after the manual reclassification. Finally, the file Profils_by_clusters.csv gathers the weighted average time series of the 18 clusters. A throughout description of the clusters and an analysis of their properties is given in the document ELMAS_data_analysis.pdf.

**Table 4:** Summary of columns in the available files.

File	Col. name	Format	Units	Description
NACE_classification	Section / Class	character		Coding of the NACE sections / classes
	Section_description / Class_description	string		Description of the NACE sections / classes
Nb_customers_time_series	Section / Class / Power_level	character		Coding of the NACE sections and classes / Level of subscribed power
	Nb_customer	float		Number of customers
Annual_energy_time_series	Power_level / Section / Class	character		Level of subscribed power / Coding of the NACE sections and classes
	Energy	float	kWh	Annual energy consumption of the groups associated with the considered time series
Mean_sd_nace_classes_time_series	Power_level / Section / Class	character		Level of subscribed power / Coding of the NACE sections and classes
	Mean / Sd	float	kWh	Average and standard deviation of the time series
Temperature	Time	character		Temporal sequence
	Temperature	float	^°^*C*	Hourly temperature at the national level
Annual_energy_weights	Power_level / Section / Class	character		Level of subscribed power / Coding of the NACE sections and classes
	Energy	float	kWh	Annual energy consumption of the wide panel of customers
Energy_consumption_per_unit_surface_area	Class / Description	character		NACE class coding / Description of the NACE class
	Energy	float	kWh	Annual energy consumption
	Surface	float	m^2^	Surface area of buildings that belong to the NACE class
	Energy_m2	float	kWh/m^2^	Annual energy consumption per unit area
Clusters_before_manual_reclassification	Power_level / Class	character		Level of subscribed power / NACE classes coding
	Cluster	int		Code of the assigned cluster
Clusters_after_manual_reclassification	Power_level / Class	character		Level of subscribed power / NACE classes coding
	Cluster	int		Code of the assigned cluster
Time_series_18_clusters	Time	character		Temporal sequence
	1 → 18	float	kWh	Weighted average of the consumption of the clusters

## Technical Validation

The quality of clustered data can be evaluated using either cluster- or load-specific criteria. The first kind of score was employed in the methods section to determine the optimal number of clusters to consider. In this section, the focus is on the analysis of criteria that characterise the load consumption.

### Consistency of the clustering

**Fig. 9:**
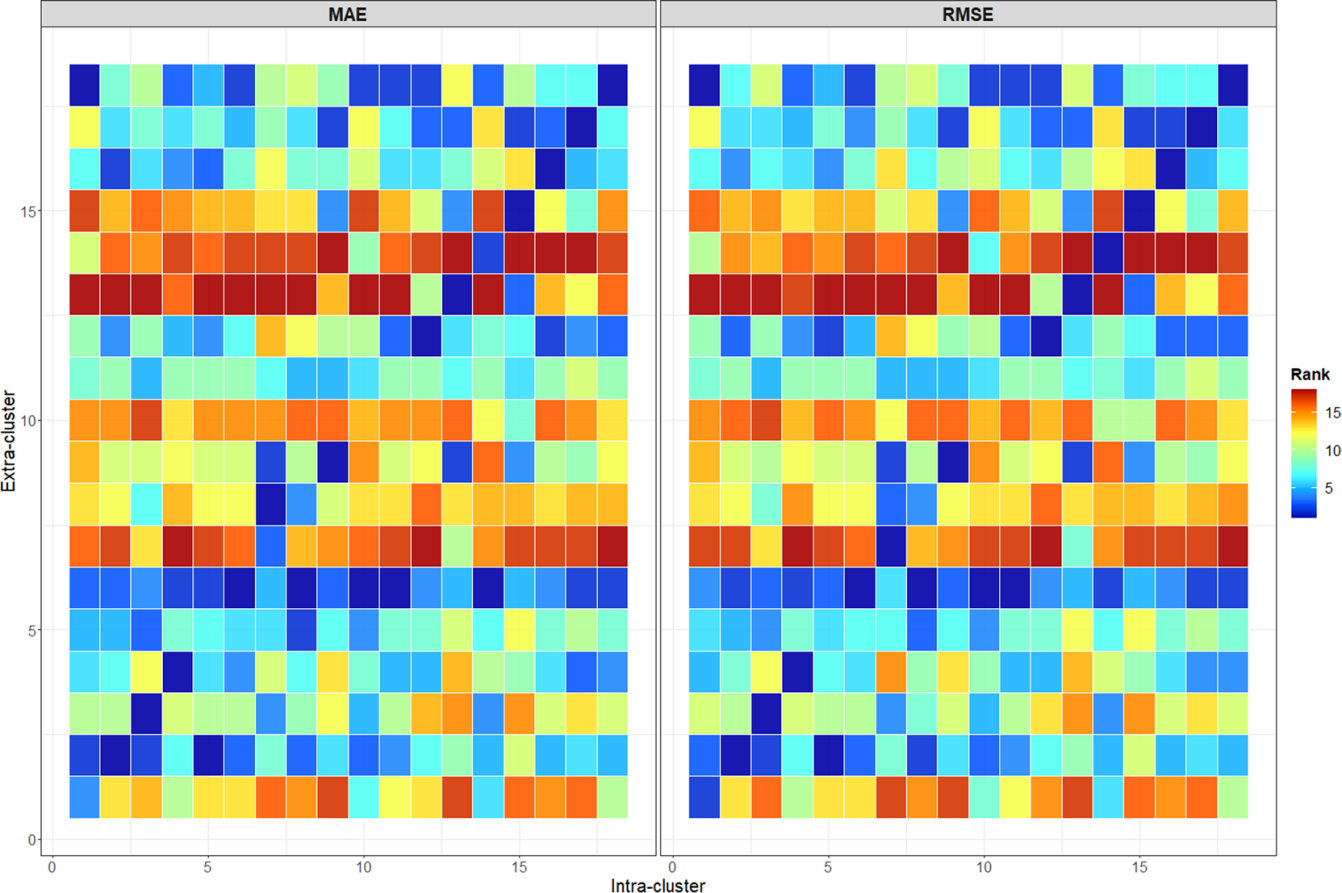
Averaged MAE and RMSE scores of the NACE classes w.r.t. the cluster they belong to (i.e. intra-cluster) and other clusters (i.e. extra-cluster). The scores are derived from the consumption time series of the NACE classes and the load profiles of the 18 clusters. For readers’ convenience, the heat-maps represent the ranking of the scores according to the extra-cluster feature: low values (i.e. blue colour) indicate the best performances, while high values correspond to low scores (i.e. red colour). For instance, the average RMSE score achieved by the NACE classes that belong to cluster 15 is the lowest when computed with the average load profiles of cluster 15. We expect scores to be the lowest for time series of NACE classes computed with the profile of the cluster they belong to; this ideal state is marked by the first diagonal. Standardised load profiles and yearly time series are considered to build these figures.

First, scores typically used in the energy modelling field are considered to evaluate the closeness of the 424 NACE classes time series with the 18 derived weighted average load profiles. To do so, the Mean Absolute Error (MAE) and Root Mean Square Error (RMSE) (Equation (6)) scores are used to measure the error in terms of consumed energy. The terms *Y*_*j*_ and *X*_*i*_ respectively represent the load profiles of the cluster *j* and the NACE class *i*, while *N*_*obs*_ is the number of temporal observations. Both scores are computed for each cluster and each NACE class, then, for convenience scores are aggregated w.r.t. to the cluster the NACE classes belong to. As a result, this approach provides for all NACE classes that belong to the same cluster, a measure in terms of MAE and RMSE of the errors within the cluster and with the other clusters. Results are gathered in Fig. 9. On the whole, the scores are the lowest when the time series of NACE classes are compared with the load profile of the cluster they belong to. This tends to validate the proposed clustering approach. However, some time series associated with the NACE classes are closer to other clusters. This is the case for time series from clusters 10 and 11 that exhibit lower scores when compared with cluster 6. For that matter, cluster 6 demonstrates a high degree of similarity with most of the NACE classes compared to other clusters such as clusters 13 and 7, which appear to be more specific.

6 $$MAE({Y}_{j},{X}_{i})=\frac{1}{{N}_{obs}}\mathop{\sum }\limits_{t=1}^{{N}_{obs}}| {\overline{Y}}_{j}^{t}-{\overline{X}}_{i}^{t}| ,\,\mathrm{and,}\,\quad RMSE({Y}_{j},{X}_{i})=\sqrt{\frac{1}{{N}_{obs}}\mathop{\sum }\limits_{t=1}^{{N}_{obs}}{({\overline{Y}}_{j}^{t}-{\overline{X}}_{i}^{t})}^{2}}$$

### Variability within clusters

Then, we compare the variability within the clusters according to two axes: (1) the clustering strategy, and (2) the temporal resolution of the load profiles.

For the first dimension, we consider either a NACE section-based classification strategy (i.e. the clusters are classified w.r.t. to the NACE sections) or the clustering structure provided by the K-means algorithm which led to 18 clusters. For each approach we define two sets that gather the NACE classes associated with each cluster: the set ${C}_{k}^{NACE}$ stores the NACE classes that belong to the NACE section *k*, while the set ${C}_{k}^{K-means}$ groups the NACE classes that are affiliated to the cluster *k* obtained from the K-means algorithm. The second axis of this analysis is related to the temporal resolution of the load profiles. Three types of curve are considered: hourly, daily, and weekly load profiles. Set *T*^*Hourly*^ gathers the ${N}_{T}^{Hour}=24$ observations associated with the hour of the day, set *T*^*Daily*^ groups the consumption of the ${N}_{T}^{Week}=7$ days of the week, while set *T*^*Weekly*^ represents the ${N}_{T}^{Week}=52$ weeks of the year. These profiles are computed for all NACE classes, and for all centroids in the two clustering strategies. For the former category, the standardised consumption of the NACE class is averaged according to the temporal resolution of the considered load profiles, while the load profiles of the centroids are derived taking into account the importance of the NACE classes in terms of annual consumed energy (Equation (7)).

7 $${\overline{Y}}_{j}^{S,T}=\frac{\sum _{k\in {C}_{j}^{S}}{\omega }_{k}\cdot {\overline{X}}_{k}^{T}}{\sum _{k\in {C}_{j}^{S}}{\omega }_{k}}$$

$$\begin{array}{l}{\overline{Y}}_{j}^{S,T}\,\,\,\mathrm{Vector\ of\ standardised\ weighted\ average\ load\ profile\ for\ cluster}\,\,j,\,(\,\mathrm{e.g.}\,\,{\overline{Y}}_{j}^{S,T}=[{\overline{y}}_{j}^{S,{t}_{1}}\cdots {\overline{y}}_{j}^{S,{t}_{24}}]\,\,\mathrm{for\ the\ hourly\ profile})\,[{\rm{\varnothing }}],\\ S\,\,\,\mathrm{The\ clustering\ strategy},\,S=\{\,\mathrm{NACE\ sections},\,\,K \mbox{-} \mathrm{means\ clusters}\},\\ T\,\,\,\mathrm{The\ temporal\ resolution\ of\ profiles},\,T=\{\,\mathrm{hourly,\ daily,\ weekly}\},\\ {C}_{j}^{S}\,\,\,\mathrm{Set\ of\ NACE\ classes\ that\ belong\ to\ cluster}\,\,j\,\,\mathrm{w.r.t.\ the\ clustering\ strategy}\,S,\\ {\omega }_{k}\,\,\,\mathrm{Annual\ energy\ consumption\ of\ the\ NACE\ class}\,\,k\,[kWh],\\ {\overline{X}}_{k}^{T}\,\,\,\mathrm{Vector\ of\ standardised\ load\ profile\ of\ the\ NACE\ class}\,\,k\\ \,\,\mathrm{for\ the\ temporal\ period}\,\,T\,[{\rm{\varnothing }}].\end{array}$$

The next step consists in computing an estimation of the dispersion -of the sample within a cluster following Equation (8) for each clustering strategy and temporal aggregation resolution. Then, results are averaged for all clusters of the weighting strategy *S* (Equation (9)), and according to the temporal dimension via Equation (10).

8 $${\Sigma }_{j}^{S,T}=\sqrt{\frac{1}{{N}_{NACE}^{{C}_{j}^{S}}}\sum _{k\in {C}_{j}^{S}}{({\overline{X}}_{k}^{T}-{\overline{Y}}_{j}^{S,T})}^{2}}$$

9 $${\Sigma }^{S,T}=\frac{1}{{N}_{{C}^{S}}}\mathop{\sum }\limits_{j=1}^{{N}_{C}}{\Sigma }_{j}^{S,T}$$

10 $${\widetilde{\sigma }}^{S,T}=\frac{1}{{N}_{T}}\sum _{t\in T}{\sigma }_{j}^{X,t}$$

$$\begin{array}{l}{N}_{NACE}^{{C}_{j}^{S}}\,\,\,\mathrm{Number\ of\ NACE\ classes\ contained\ in\ the\ cluster}\,j\\ \,\,\mathrm{obtained\ with\ the}\,\,S\,\,\mathrm{clustering\ strategy},\\ {\Sigma }_{j}^{S,T}\,\,\,\mathrm{Vector\ of\ the\ standard\ deviation\ of\ the\ NACE\ profiles\ that\ belong\ to\ the}\,\\ \,\,\mathrm{cluster}\,j\,\mathrm{w.r.t.\ to\ the\ centroid\ of\ that\ cluster}\,\,[{\rm{\varnothing }}],\\ {\Sigma }^{S,T}\,\,\,\mathrm{Vector\ of\ the\ standard\ deviation\ for\ each\ instance\ of\ the\ load\ profile}\\ \,\mathrm{for\ clustering\ strategy}S\,[{\rm{\varnothing }}],\\ {\widetilde{\sigma }}^{S,T}\,\,\,\mathrm{Average\ standard\ deviation\ within\ clusters\ for\ clustering\ strategy}\,S\\ \,\,\mathrm{and\ temporal\ resolution}\,\,T\,[{\rm{\varnothing }}],\\ {N}_{{C}^{S}}\,\mathrm{Number\ of\ clusters\ in\ the\ clustering\ strategy}\,\,S,\\ {N}_{T}\,\mathrm{Number\ of\ temporal\ observations\ associated\ with\ resolution}\,\,T.\end{array}$$

The variability within clusters is shown in Table 5. We observe that for the three temporal resolutions analysed, the K-means-derived clustering leads to the best average standard deviation within clusters. This demonstrates that the proposed approach provides more compact clusters than those that would have been obtained from the NACE-based classification.

**Table 5:** Average standard deviation within clusters w.r.t. the clustering strategies and the temporal aggregation of the load profiles.

Temporal resolution	NACE section-based clustering	K-means-based clustering
Hourly	170.93	166.41
Daily	27.30	27.04
Weekly	463.68	428.25

Standardised inputs are considered.

## Usage Notes

This section discusses the applicability and the limitations of the dataset.

First, as the initial dataset of electricity consumption was collected during 2018, the latter is free from any impacts associated with the COVID outbreak (e.g. reduction of professional activities). This suggests that new practices such as teleworking are not present in the proposed load profiles. Therefore, some profiles related to office work may be outdated.

Potential users should be aware that the proposed profiles are climate-zone-dependent due to the temperature-sensitivity of some business sectors (e.g. through the use of electric heating or air conditioning devices). Thus, their use should be restricted to climates similar to that of France, or appropriate care should be taken. For this purpose, observations of the temperature at the national level are given.

The generated load profiles are provided in the form of standardised values. Relevant information, such as the mean, standard deviation, and annual energy consumption associated with each NACE class, is provided to allow the user to perform de-standardisation. The areas associated with some NACE classes are also supplied for scaling purposes.

## Data Availability

The code used to cluster the time series is not publicly available because, in the absence of input data, it can not be executed. However, special attention has been paid to provide a detailed description of the clustering approach for transparency in this article. In addition, the source code used in R^29^ to perform the data analysis is provided with the ELMAS dataset. All scripts have been tested working as of 19/03/2023 on a machine running Windows 10, and using R version 4.1.0 (2021-05-18). The required packages to run the scripts are detailed in the code, and the purpose of each script is defined in its header.
